# Evaluating the disease progression of pediatric bicuspid aortic valve patients using 4D flow MRI data

**DOI:** 10.1186/1532-429X-18-S1-P170

**Published:** 2016-01-27

**Authors:** Michael J Rose, Kelly B Jarvis, Alex J Barker, Susanne Schnell, Bradley D Allen, Joshua D Robinson, Michael Markl, Cynthia K Rigsby

**Affiliations:** 1 grid.413808.60000000403882248Medical Imaging, Ann & Robert H. Lurie Children's Hospital of Chicago, Chicago, IL USA; 2 grid.465264.7Radiology, Northwestern University, Chicago, IL USA; 3 grid.465264.7Biomedical Engineering, Northwestern University, Chicago, IL USA; 4 grid.413808.60000000403882248Pediatric Cardiology, Ann & Robert H. Lurie Children's Hospital of Chicago, Chicago, IL USA; 5 grid.465264.7Pediatrics, Northwestern University, Chicago, IL USA

## Background

Understanding the natural history of bicuspid aortic valve (BAV) disease from childhood through adulthood may aid in determining if and when these patients are at risk for disease progression. 4D flow MRI can be used to visualize blood flow patterns in BAV patients and can be used to assess valvular function. In this study, we track the progression of pediatric BAV disease by assessing flow pattern and velocity changes over the course of two 4D flow MRI exams separated by at least 7 months between baseline and follow-up scan.

## Methods

For this retrospective IRB-approved study, we reviewed 11 pediatric patients (3 females) with BAV who underwent initial (age = 13 ± 6 (1-20) years) and follow up (age=15 ± 6.0 (2-22) years) 4D flow MRI studies as part of a clinical cardiac MRI examination. All MRI studies were performed at 1.5 T with spatial resolution = 1.23-3.46 x 1.13-2.5 x 1.2-3.0 mm^3^, temporal resolution 37.6-44 ms, TE/TR/FA = 2.3-2.8 ms/4.7-5.1 ms/15° and velocity sensitivity = 150-400 cm/s. 4D flow data were preprocessed to reduce noise and artifacts caused by velocity aliasing and phase offset errors (Maxwell terms, eddy currents). 3D PCMR angiograms were computed from 4D flow and used to obtain a 3D segmentation of the thoracic aorta (Mimics, Materialise, Belgium). The 4D flow velocity field was masked by the 3D segment and used to generate velocity maximum intensity projections (MIPs) using an in-house tool. Peak velocities in the ascending aorta (AAo) were determined from the MIPs using region of interest (ROI) analysis. Aortic root diameters and Z-scores as recorded from EchoIMS were measured from either systolic 3D gradient echo MRA or steady state free precession cine MRI during the same MR assessment as the 4D flow study. Peak systolic AAo velocities, aortic root Z-scores, and flow patterns using the systolic MIP image from the first CMR study were compared to the second study for each patient.

## Results

**Figure 1:**
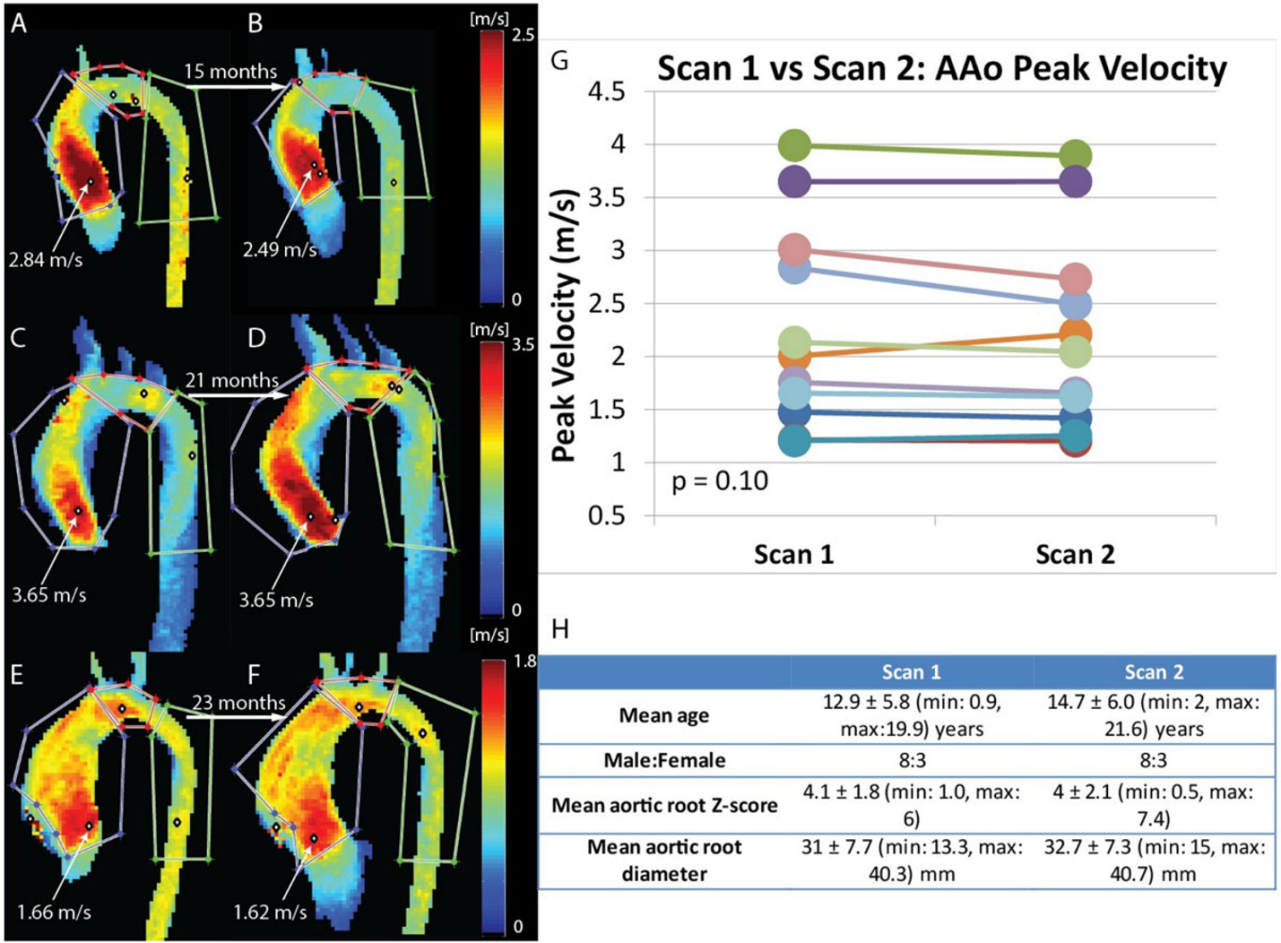
**A)-F) Velocity MIPs derived from aortic 4D flow data**. The white points outlined in black indicate the peak velocity found in each of the 3 regions of interest: ascending aorta (AAo), aortic arch and descending aorta. **A)** and **B)** are the first (age: 19 years) and second scans of the same patient, respectively, 15 months apart. **C)** and **D)** are the first (age: 20 years) and second scans of the same patient, respectively, 21 months apart. **E)** and **F)** are the first (age: 14 years) and second scans of the same patient, respectively, 23 months apart. **G)** Plot of the differences in AAo peak velocities between the first and second scan for all patients. The Wilcoxon signed-rank sum test (p =0.10) was used to test the significance of the difference between scan 1 and scan 2 AAo peak velocities. **H)** Patient Characteristics.

The 4D flow MRI exams were separated by a mean of 21 ± 11 (7-37) months. No significant difference was seen in mean systolic AAo peak velocities (2.27 ± 0.97 vs 2.20 ± 0.92 m/s, p = 0.10) or aortic root Z-scores (4.1 ± 1.8 vs. 4 ± 2.1, p = 0.48) between the 1^st^ and 2^nd^ study. As shown for 3 pediatric BAV patients in figure 1A-F, flow patterns between baseline and follow-up CMR studies were remarkably similar for each patient, but substantially different between patients.

## Conclusions

There were no significant changes in AAo peak systolic velocity or aortic root Z-scores and flow patterns were stable between the studies. This suggests that the small progression of the disease in this short-term follow-up study (stable z-scores) is accompanied by highly reproducible aortic flow 4D patterns indicating the robustness of 4D flow MRI to visualize and quantify patient specific aortic hemodynamics. 4D flow MRI can thus provide a reliable patient specific baseline of aortic hemodynamics which may be important to identify changes in aortic blood flow during long-term follow-up.

